# Dental Therapeutics

**Published:** 1867-12

**Authors:** Wm. O. Kulp

**Affiliations:** Muscatine, Iowa


### DENTAL THERAPEUTICS.

AT the last meeting of the American Dental Association, held at Cincinnati, Ohio, there was a committee appointed and instructed to prepare a report on the above subject, and present it at the next meeting of the association.

As a member of this committee I respectfully ask gentlemen of the profession of all parts of the country to communicate either through the Dental Journals or directly to me, any thing they have new on this subject; also, to give their experience with old remedies; give as far as possible your opinion of the *modus operandi* of the remedies you mention. Communications from gentlemen of the medical profession on this subject will be gratefully received.

This committee desires to make as extended and thorough a research on this subject as possible; but the time is short! So brethren, if you will assist us, we will promise you a summary of all that is known by the profession on this very important subject. On behalf of Committee,

WM. O. KULP, D. D. S.

December 26th, 1867. Muscatine, Iowa.

